# Effect of esketamine-based patient-controlled intravenous analgesia on postoperative pain and quality of recovery after video-assisted thoracoscopic lobectomy: A prospective, double-blind, randomized controlled trial

**DOI:** 10.1371/journal.pone.0340864

**Published:** 2026-01-27

**Authors:** Ruirui Bi, Jiqiang Zhang, Ruijuan Liu, Lijuan Li, Yuxi Su, Mengjun Xu, Wenjun Yan

**Affiliations:** 1 Department of Anesthesiology, Nanjing Drum Tower Hospital, Affiliated Hospital of Medical School, Nanjing University, Nanjing, Jiangsu Province, China; 2 The First Clinical Medical College of Gansu University of Chinese Medicine (Gansu Provincial Hospital), Lanzhou, Gansu Province, China; 3 Department of Anesthesiology, Gansu Provincial Hospital, Lanzhou, Gansu Province, China; Monash University, ETHIOPIA

## Abstract

**Objective:**

This double-blinded randomized study aimed to investigate the effects of esketamine-based patient-controlled intravenous analgesia (PCIA) on postoperative analgesia and quality of recovery in patients undergoing video-assisted thoracoscopic (VATS) lobectomy.

**Methods:**

Patients undergoing selective VATS lobectomy were enrolled and randomly assigned (1:1) to receive PICA with 1.5 mg/kg esketamine (group K) or 1.5 mg/kg sufentanil (group S). Pain intensity was evaluated using the short-form of the McGill Pain Questionnaire (SF-MPQ) and the visual analog scale (VAS). The primary endpoint was the SF-MPQ score of patients on postoperative day 1.

**Results:**

Between December 2021 and May 2022, 84 eligible patients received the allocated treatment, with 80 patients (40 per group) ultimately included in the analysis. The total SF-MPQ score in group K was lower than that in group S on postoperative day 1 (*P* < 0.001) and day 2 (*P* < 0.001). Additionally, the VAS-rest, VAS-movement and patients’ depression-related scores in group K were all significantly lower than those in group S on postoperative day 1 (*P* = 0.012, *P* = 0.008 and *P* = 0.009, respectively) and day 2 (all *P* < 0.001), whereas the postoperative recovery quality was significantly higher in group K than that in group S on postoperative days 1 and 2 (both *P* < 0.001). A lower incidence of total adverse events (AEs) was observed in group K than in group S (15% *vs.* 35%, *P* = 0.039).

**Conclusion:**

The use of 1.5 mg/kg esketamine in PCIA for postoperative analgesia in patients undergoing VATS lobectomy showed a promising analgesic effect and improved perioperative depression and postoperative recovery quality, with no severe AEs observed.

## Introduction

Postoperative pain is the most prevalent and serious complication in patients undergoing thoracic surgery [1], with more than 90% of patients experiencing this pain [2]. In addition, 59% of patients have moderate or severe pain after video-assisted thoracoscopic surgery (VATS) [3]. Inadequate management of postoperative analgesia predisposes to an increase in pulmonary complications such as postoperative pneumonia, atelectasis, and hypoxia, thereby increasing morbidity and mortality, as well as hindering patient recovery [4–6]. Therefore, adequate analgesia is of vital importance for early postoperative activities, which restore lung function as early as possible.

N-methyl-D-aspartate (NMDA) antagonists have been proven to provide good analgesic effects and been recommended as perioperative analgesic drugs [7–9]. Esketamine, a NMDA antagonist, exerts both analgesic and antidepressant effects. It has been shown that esketamine, when applied intraoperatively, exhibits a good analgesic effect [10]. However, little attention has been paid to the influence of intravenous esketamine on postoperative pain in patients undergoing VATS.

This study aimed to assess the effects of patient-controlled intravenous analgesia (PCIA) with esketamine on postoperative analgesia and quality of recovery in patients undergoing VATS lobectomy, and to explore the feasibility of opioid-free analgesia with PCIA in the postoperative setting.

## Methods

### Study design

This prospective, randomized, double-blind, controlled trial was conducted at Gansu Provincial Hospital in China. This study was approved by the Medical Ethics Committee of Gansu Provincial Hospital (No. 2021-277), and was registered with the Chinese Clinical Trial Registry (Registration no. ChiCTR2100054418). All patients provided written informed consent.

### Patients

The inclusion criteria were as follows: (1) patients scheduled for VATS lobectomy; (2) aged 18–65 years; (3) American Society of Anesthesiologists (ASA) physical status of I–II; (4) body mass index (BMI) of 18–30 kg/m^2^; (5) clear consciousness, with normal reading comprehension, vision, and hearing; (6) no history of mental illness or cognitive impairment; and (7) voluntary participation in this study, with signed informed consent.

The exclusion criteria were as follows: (1) allergies or contraindications to drugs and adjuvants used in the perioperative period; (2) participation in other clinical studies within 3 months before this study; (3) pregnancy or alcohol abuse; (4) a history of psychiatric or cognitive disorders, with recent use of psychotropic drugs; and (5) an inability to communicate with the physician.

The discontinuation of participation criteria were as follows: (1) interruption of PCIA because of serious adverse events or other reasons; (2) the emergence of other illnesses during the trial; (3) poor compliance or failure to provide critical assessment data.

### Randomization and blinding

The patients were randomly assigned in a 1:1 ratio to receive esketamine (group K) or sufentanil (group S) according to a random number table generated by IBM SPSS Statistics for Windows, version 25.0 (IBM Corp., Armonk, N.Y., USA). The allocations were concealed and kept in sealed envelopes. The postoperative PCIA pumps were uniformly prepared and installed by an analgesic management team according to the envelopes. The analgesic management team was not involved in randomization and data collection. All PCIA pumps were identical in appearance and infusion parameters. Data collection was performed by two blinded anesthesia nurses, one for paper data, and the other for blood samples data. All surgeons, anesthesiologists, surgery nurses, patients and data collectors were blinded to treatment assignment.

### General anesthesia

Upon entering the operating room, patients were established with venous access and monitored for vital signs, including noninvasive blood pressure (NIBP), pulse oxygen saturation (SpO_2_), five-lead electrocardiogram (ECG), and end-expiratory carbon dioxide partial pressure (PetCO_2_). General anesthesia was induced sequentially by intravenous administration of midazolam 0.05 mg/kg, propofol 2 mg/kg, sufentanil 0.5 μg/kg, and cisatracurium 0.2 mg/kg. Endotracheal intubation was performed 3 min after administration, and the respiration was controlled by a ventilator (respiratory rate = 12 beats/min, tidal volume = 8 mg/kg). General anesthesia was maintained with propofol 6−8 mg/kg/hour, remifentanil 0.1–0.2 μg/kg/hour, and dexmedetomidine 0.2–0.7 μg/kg/hour, supplemented by intermittent administration of cisatracurium 0.1 mg/kg every 30 min. After the surgery, patients were transferred to the post-anesthesia care unit (PACU) and extubated.

### Interventions

All patients were given PCIA in the PACU. The storage bag of the PCIA contained 150 ml of solution, with a background infusion rate of 2 ml/hour, a bolus dose of 2 ml, and a lock time of 15 min. The total 150 ml dose of PCIA in group K was esketamine 1.5 mg/kg, flurbiprofen axetil 250 mg, metoclopramide 50 mg, dexmedetomidine 1 μg/kg, and appropriate normal saline; while the total 150 ml dose of PCIA in group S, was sufentanil 1.5 μg/kg, flurbiprofen axetil 250 mg, metoclopramide 50 mg, dexmedetomidine 1 μg/kg, and appropriate normal saline.

If the visual analog scale at rest (VAS-rest) was ≥ 4, patients could press the PCIA button to receive a bolus dose. If the pain was not relieved after a bolus dose or the VAS-rest was ≥ 7, patients received one oral tablet of oxycodone and acetaminophen as rescue analgesia.

### Assessment

All outcome measures were performed at 9: 00–10: 00 am before operation, on postoperative day 1 (POD 1) and postoperative day 2 (POD 2) by an independent observer blinded to the entire study process. The primary outcomes of this study were patients’ pain conditions. Patients’ pain conditions were evaluated using the short-form of the McGill Pain Questionnaire (SF-MPQ), which consists of 15 items designed to evaluate sensory and affective of pain [11]. The VAS-rest score and the VAS-movement score were adopted to measure the postoperative pain intensity of patients at rest and during coughing or movement, respectively. The total SF-MPQ scores were the sum of the sensory score, affective score, VAS-rest score, and present pain intensity (PPI), because the patient was required to be in a quiet state when being assessed.

This study also explored patients’ perioperative depression and quality of life. Patients’ perioperative depression and quality of life were evaluated using the Patient Health Questionnaire-9 (PHQ-9) and EuroQol-5 Dimension (EQ-5D) [12,13]. Other outcomes included time to out-of-bed mobilisation, use of rescue analgesics, and the incidence of adverse events (AEs) during the postoperative analgesia period. In cases of serious AEs, PCIA was discontinued immediately, and the corresponding AEs were managed. Patient and observer satisfaction with analgesia was collected on POD 2 using a five-level Likert scale, with 1 point representing very dissatisfied and 5 points representing very satisfied.

Furthermore, blood samples from some patients were randomly collected for the measurement of interleukin-6 (IL-6), tumor necrosis factor-α (TNF-α), and brain-derived neurotrophic factor (BDNF) levels in both groups at three points: 12 h before surgery, as well as 24 h and 48 h after surgery. The blood samples were analyzed using an enzyme-linked immunosorbent assay (ELISA) kit (Meimian Biotechnology, Yancheng, Jiangsu, China).

To ensure data security, the paper-based data were sealed and archived in the trial hospital’s database after entry into the statistical analysis system. The serum samples were destroyed by the laboratory after the data were collected.

### Statistical analysis

According to the results of our preliminary study, the 24-h VAS-movement score was 2.6 ± 0.8 in patients receiving sufentanil-based PCIA, and was 2.0 ± 1.0 in those receiving esketamine-based PCIA. Assuming α = 0.05 (two-tailed) and power 80%, the required sample size for each group was calculated to be 37 patients. The sample size calculation was performed using PASS software (version 21.0.3, NCSS, LCC, Kaysville, UT, United States).

Normally distributed variables were presented as mean (SD) and analyzed using a two-sample independent t-test. Non-normally distributed variables were reported as median (IQR) and analyzed using the Mann–Whiney U test. Categorical variables were expressed as number (percentages) and compared using the Chi-square or Fisher’s exact test. A *P*-value of < 0.05 (two-tailed) was considered statistically significant. Statistical analyses were conducted using IBM SPSS Statistics, version 25.

The SF-MPQ, PHQ-9, and EQ-5D scores in this study were non-normally distributed variables, and the Mann-Whitney U test was used to compare the results between the two patient groups at the same time points, and within-group comparisons across different time points were made using Friedman’s test and Wilcoxon signed rank-sum test, with Bonferroni correction applied.

For missing data, when the proportion of missing data was less than 5%, the deletion method was used to handle missing data. When the proportion of missing data was greater than 5%, multiple imputation was used to handle the data.

## Results

**Fig 1:**
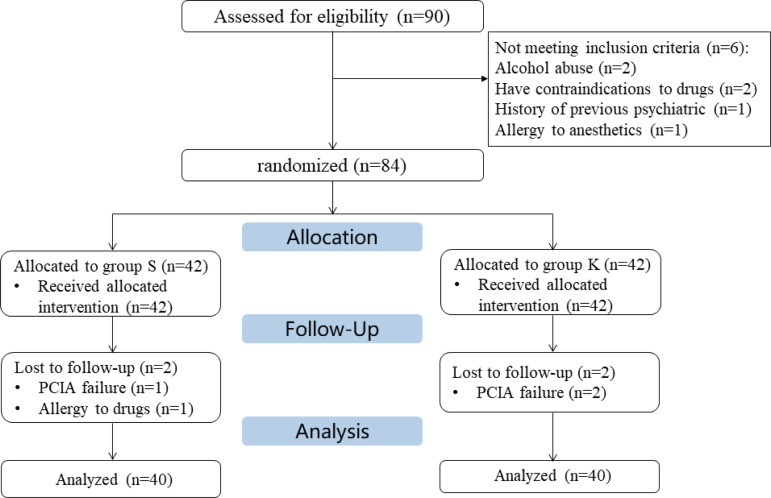
CONSORT flow diagram.

Between December 2021 and May 2022, a total of 90 patients were screened for eligibility in this study, of which six patients were excluded due to alcohol abuse (n = 2), contraindications to drugs (n = 2), a history of psychiatric disorders (n = 1), and allergies to anesthetics (n = 1), respectively. A total of 84 eligible patients were randomly assigned to either group K or group S, with 42 patients in each group. Of them, three patients did not complete the PCIA (two in group K and one in group S), and one in group S experienced an allergic reaction during analgesia. Finally, 40 patients per group were included in the efficacy and safety analyses. The CONSORT flowchart for study participants is presented in Fig 1. The baseline characteristics of patients were well balanced between the groups (*P*> 0.05; Table 1).

**Table 1:** Baseline characteristics.

	Group S (*n* = 40)	Group K (*n* = 40)	*P* value
Age (years), median (IQR)	52.5 (45.0–59.0)	52.0 (47.25–59.0)	0.847
Sex, *n* (%)			0.485
Male	27 (67.5)	24 (60)	
Female	13 (32.5)	16 (40)	
BMI (kg/m^2^), mean (SD)	22.71 (2.82)	22.32 (2.65)	0.522
ASA level, *n* (%)			0.531
Ⅰ	7 (17.5)	5 (12.5)	
Ⅱ	33 (82.5)	35 (87.5)	

Mann-Whitney U test was used to compare the Age between two groups. Chi-square test was used to compare the Sex and ASA level between two groups. Two-sample independent t-test was used to compare the BMI between two groups. Group K, esketamine group; Group S, sufentanil group. BMI, body mass index; ASA, American Society of Anesthesiologists.

### Changes in the analgesic effect in the two groups

**Fig 2:**
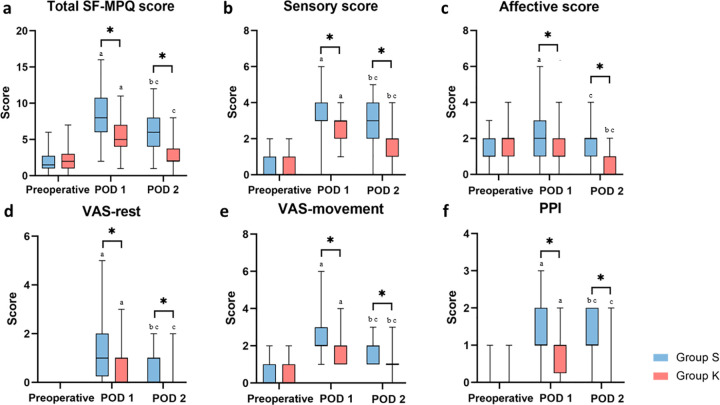
Perioperative SF-MPQ score of patients receiving PCIA. Mann-Whitney U test was used to compare the results between two groups of patients at the same time points. Wilcoxon signed rank-sum test with Bonferroni correction were used for within-group comparisons. ^*^Group K *versus* Group S (*P*< 0.05). ^a^ The within-group difference between the pre-operation and POD 1 was significant, with *P*_(Bonferroni correction)_ < 0.05/3 = 0.016. ^b^ The within-group difference between the pre-operation and POD 2 was significant, with *P*_(Bonferroni correction)_ < 0.05/3 = 0.016. ^c^ The within-group difference between the POD 1and POD 2 was significant, with *P*_(Bonferroni correction)_ < 0.05/3 = 0.016. Group K, esketamine group; Group S, sufentanil group. SF-MPQ, Short-Form McGill Pain Questionnaire; POD, postoperative day; VAS, visual analog scale; PPI, present pain intensity.

No significant differences were observed in the total SF-MPQ score, sensory score, affective score, VAS-rest score, VAS-movement score, and present pain intensity (PPI) score between the two groups prior to surgery (all *P*> 0.05). In contrast, the total SF-MPQ score in group K was significantly lower than that in group S on POD 1 (*P* < 0.001) and POD 2 (*P*< 0.001). Furthermore, the sensory and affective scores of the SF-MPQ in group K were significantly lower than those in group S on POD 1 and POD 2 (sensory score: *P*= 0.001, *P*< 0.001; affective score: *P*= 0.007, *P* < 0.001, respectively). The VAS-rest score, VAS-movement score, and PPI of the SF-MPQ in group K were also significantly lower than those in group S on POD 1 (*P*= 0.012, *P*= 0.008, and *P*= 0.003, respectively) and POD 2 (all *P*< 0.001; Fig 2 and Table 2).

**Table 2:** Perioperative SF-MPQ score of patients receiving PCIA.

Variables, median (IQR)	Group S (*n* = 40)	Group K (*n* = 40)	*z*	*P* value
Total SF-MPQ score				
Pre-operation	1.5 (1.00–2.75)	2.0 (1.00–3.00)	−1.234	0.217
POD 1	8.0 (6.00–10.75)^a^	5.0 (4.00–7.00)^a^	−3.650	<0.001^*^
POD 2	6.0 (4.00–8.00)^b c^	2.0 (2.00–3.75)^c^	−5.686	<0.001^*^
Sensory score				
Pre-operation	0.0 (0.00–1.00)	0.0 (0.00–1.00)	−0.267	0.790
POD 1	3.0 (3.00–4.00)^a^	3.0 (2.00–3.00)^a^	−3.285	0.001^*^
POD 2	3.0 (2.00–4.00)^b c^	2.0 (1.00–2.00)^b c^	−3.63	<0.001^*^
Affective score				
Pre-operation	1.0 (1.00–2.00)	2.0 (1.00–2.00)	−1.379	0.168
POD 1	2.0 (1.00–3.00) ^a^	1.0 (1.00–2.00)	−2.679	0.007^*^
POD 2	2.0 (1.00–2.00)^c^	0.0 (0.00–1.00)^b c^	−5.223	<0.001^*^
VAS-rest				
Pre-operation	0.0 (0.00–0.00)	0.0(0.00–0.00)	0.000	1.000
POD 1	1.0 (0.25–2.00) ^a^	1.0(0.00–1.00)^a^	−2.51	0.012^*^
POD 2	1.0 (0.00–1.00)^b c^	0.0(0.00–0.00)^c^	−4.528	<0.001^*^
VAS-movement				
Pre-operation	0.0 (0.00–1.00)	0.0 (0.00–1.00)	−0.144	0.885
POD 1	2.0 (2.00–3.00) ^a^	2.0 (1.00–2.00) ^a^	−2.66	0.008^*^
POD 2	2.0 (1.00–2.00)^b c^	1.0 (1.00–1.00)^b c^	−5.269	<0.001^*^
PPI				
Pre-operation	0.0 (0.00–0.00)	0.0 (0.00–0.00)	−0.459	0.646
POD 1	1.0 (1.00–2.00) ^a^	1.0 (0.25–1.00) ^a^	−2.992	0.003^*^
POD 2	1.0 (1.00–2.00)^b c^	0.0 (0.00–0.00)^c^	−5.352	<0.001^*^

Data are median (IQR). Mann-Whitney U test was used to compare the results between two groups of patients at the same time points. Wilcoxon signed rank-sum test with Bonferroni correction were used for within-group comparisons. Group K, esketamine group; Group S, sufentanil group; IQR, interquartile range; SF-MPQ, Short-Form McGill Pain Questionnaire; POD, postoperative day; VAS, visual analog scale; PPI, present pain intensity.

* The difference between the two groups was significant, with a *P* value of < 0.05.

^a^ The within-group difference between the pre-operation and POD 1 was significant, with a *P*_(Bonferroni correction)_ value of < 0.05/3 = 0.016.

^b^ The within-group difference between the pre-operation and POD 2 was significant, with a *P*_(Bonferroni correction)_ value of < 0.05/3 = 0.016.

^c^ The within-group difference between the POD 1 and POD 2 was significant, with a *P*_(Bonferroni correction)_ value of < 0.05/3 = 0.016.

The median total PCIA dosage for postoperative analgesia was 88.5 (IQR 78.5–99) ml in group K, which was significantly lower than the 97 (IQR 88.25–113.5) ml observed in group S (*P*= 0.014). Moreover, the median total number of rescue analgesic administrations required in group K was 0 (IQR 0–1), which was significantly fewer than the 1 (IQR 0–2) recorded in group S (*P*= 0.004). Patient and observer satisfactions with analgesia in group K were also significantly higher than those in group S (*P*= 0.001 and *P*< 0.001; Table 3).

**Table 3:** PCIA parameters and postoperative satisfaction.

Variables	Group S (*n* = 40)	Group K (*n* = 40)	*z*	*P* value
PCIA dosage (mL)				
POD 1	49.5 (43.25–54.00)	45.5 (42.00–49.75)	−1.975	0.048^a^
POD 2	97.0 (88.25–113.50)	88.5 (78.50–99.00)	−2.455	0.014^a^
Number of rescue analgesia				
POD 1	0.0 (0.00–1.00)	0.0 (0.00–0.00)	−2.122	0.034^a^
POD 2	1.0 (0.00–2.00)	0.0 (0.00–1.00)	−2.894	0.004^a^
Patients’ satisfaction	4.0 (3.0–5.0)	5.0 (4.0–5.0)	−3.34	0.001^a^
Observer’s satisfaction	4.0 (3.0–5.0)	5.0 (4.0–5.0)	−3.804	<0.001^a^

Data are median (IQR). Mann-Whitney U test was used to compare the results between two groups of patients at the same time points. Group K, esketamine group; Group S, sufentanil group. IQR, interquartile range; POD, postoperative day.

^a^ The difference between the two groups was significant, with a *P* value of < 0.05.

In group S, the total SF-MPQ scores on POD 1 and POD 2 were significantly higher than the preoperative score (both *P*_(Bonferroni correction)_ < 0.001), with the score on POD 1 also being significantly higher than that on POD 2 (*P*_(Bonferroni correction)_ < 0.001). The sensory scores on POD 1 and POD 2 were significantly higher than the preoperative score in group S (both *P*_(Bonferroni correction)_ < 0.001), and the score on POD 1 was significantly higher than that on POD 2 (*P*_(Bonferroni correction)_ = 0.001). The affective score on POD 1 was significantly higher than the preoperative score (*P*_(Bonferroni correction)_ = 0.001), and also significantly higher than that on POD 2 (*P*_(Bonferroni correction)_ = 0.002), but there was no significant difference between the affective scores on POD 2 and the preoperative day in group S (*P*_(Bonferroni correction)_ = 0.036). Additionally, the VAS-rest, VAS-movement, and PPI scores on POD 1 and POD 2 were significantly higher than their respective preoperative scores in group S (all *P*_(Bonferroni correction)_ < 0.001), with the scores on POD 1 being significantly higher than those on POD 2 (*P*_(Bonferroni correction)_ < 0.001; Fig 2 and Table 2).

In group K, the total SF-MPQ score on POD 1 was significantly higher than the scores on preoperative day and POD 2 (both *P*_(Bonferroni correction)_ < 0.001), with no significant difference between the preoperative day and POD 2 (*P*_(Bonferroni correction)_ = 0.113). The sensory scores on POD 1 and POD 2 were significantly elevated compared to the preoperative scores in group K (both *P*_(Bonferroni correction)_ < 0.001), and the score on POD 1 was significantly higher than that on POD 2 (*P*_(Bonferroni correction)_ < 0.001). No significant difference was observed in the affective scores of group K on POD 1 and prior to surgery (*P*_(Bonferroni correction)_ = 0.274), but the affective score on POD 2 was significantly lower than those on preoperational day and on POD 1 (both *P*_(Bonferroni correction)_ < 0.001). There was no significant difference between the VAS-rest scores on POD 2 and the preoperative day in group K (*P*_(Bonferroni correction)_ = 0.034), while the VAS-rest score on POD 1 was significantly higher than those on both the preoperative day and POD 2 (both *P*_(Bonferroni correction)_ < 0.001). The VAS-movement scores on POD 1 and POD 2 were also significantly higher than the preoperative score in group K (both *P*_(Bonferroni correction)_ < 0.001), with the score on POD 1 being significantly higher than that on POD 2 (*P*_(Bonferroni correction)_ < 0.001). The PPI of SF-MPQ in group K on POD 1 was significantly higher than those before surgery and on POD 2 (both *P*_(Bonferroni correction)_ < 0.001), while no significant difference was noted between the preoperative day and POD 2 (*P*_(Bonferroni correction)_ = 0.132; Fig 2 and Table 2).

### Changes in the recovery quality in the two groups

**Fig 3:**
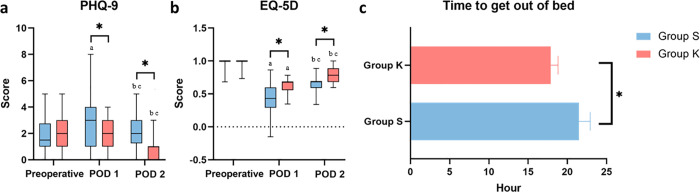
Perioperative PHQ-9 score and postoperative recovery quality of patients receiving PCIA. Mann-Whitney U test was used to compare the results between two groups of patients at the same time points. Wilcoxon signed rank-sum test with Bonferroni correction were used for within-group comparisons. ^*^Group K *versus* Group S (*P*< 0.05). ^a^ The within-group difference between the pre-operation and POD 1 was significant, with *P*_(Bonferroni correction)_ < 0.05/3 = 0.016. ^b^ The within-group difference between the pre-operation and POD 2 was significant, with *P*_(Bonferroni correction)_ < 0.05/3 = 0.016. ^c^ The within-group difference between the POD 1and POD 2 was significant, with *P*_(Bonferroni correction)_ < 0.05/3 = 0.016. Group K, esketamine group; Group S, sufentanil group. PCIA, patient-controlled intravenous analgesia; POD, postoperative day.

No significant difference was found in the total PHQ-9 score between the two groups prior to surgery (*P*= 0.337), while the PHQ-9 scores in group K were significantly lower than those in group S on POD 1 and POD 2 (*P*= 0.009 and *P*< 0.001, respectively; Fig 3a). Similar, no significant difference was found in the preoperative EQ-5D score between the two groups (*P*= 0.172), but the EQ-5D scores in group K were significantly higher than those in group S on POD 1 and POD 2 (both *P*< 0.001; Fig 3b). Additionally, the median time to out-of-bed mobilisation in group K was shorter than that in group S, with a significant difference (*P*< 0.001; Fig 3c and S1 Table).

In group S, the PHQ-9 scores on POD 1 and POD 2 were significantly higher than the preoperative score (*P*_(Bonferroni correction)_ = 0.001 and *P*_(Bonferroni correction)_ = 0.016, respectively), and the score on POD 1 was significantly higher than that on POD 2 (*P*_(Bonferroni correction)_ = 0.014; Fig 3a). The EQ-5D scores on POD 1 and POD 2 were significantly lower than the preoperative scores (both *P*_(Bonferroni correction)_ < 0.001), and the score on POD 1 was significantly fewer than that on POD 2 (*P*_(Bonferroni correction)_ < 0.001; Fig 3b and S1 Table).

In group K, there was no significant difference in the PHQ-9 scores before surgery and on POD 1 (*P*_(Bonferroni correction)_ = 0.513), while the score on POD 2 was significantly lower than the preoperative and POD 2 scores (both *P*_(Bonferroni correction)_ < 0.001; Fig 3a). Similarly, the EQ-5D scores on POD 1 and POD 2 were significantly lower than the preoperative score (both *P*_(Bonferroni correction)_ < 0.001), with the score on POD 1 being significantly fewer than that on POD 2 in group K(*P*_(Bonferroni correction)_ < 0.001; Fig 3b and S1 Table).

### Safety analysis in the two groups

The incidence of total AEs in group K were significantly lower than those in group S (15.0% vs. 35.0%; *P*= 0.039). The incidence of nausea and vomiting in group K was also significantly lower than in group S (10.0% vs. 32.5%; *P*= 0.014), whereas no significant difference was found in the occurrences of sedation and nightmare between the two groups (*P*> 0.05; Table 4). However, one patient in group K had mild sedation during postoperative analgesia, with an Observer’s Assessment of Alertness/Sedation (OAA/S) score of 4.

**Table 4:** Postoperative adverse events of patients receiving PCIA.

Events, *n* (%)	Group S (*n* = 40)	Group K (*n* = 40)	x^2^	*P* value
Nausea and vomiting	13 (32.5%)	4 (10.0%)	6.05	0.014^a^
Sedation	0 (0.0%)	1 (2.5%)	—	1.000^b^
Nightmare	1 (2.5%)	1 (2.5%)	—	1.000^b^
Total	14 (35.0%)	6 (15.0%)	4.267	0.039^a^

Chi-square test was used to compare the nausea and vomiting and total adverse events between two groups. Fisher’s exact test was used to compare the Sedation and Nightmare between two groups. Group K, esketamine group; Group S, sufentanil group.

^a^The difference between the two groups was significant, with a *P* value of < 0.05.

^b^Means Fisher’s exact test.

### Changes in the levels of IL-6, TNF-α, and BDNF in the peripheral blood

**Fig 4:**
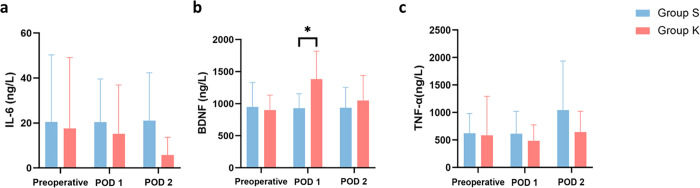
Perioperative serum IL-6/TNF-α/BDNF levels of patients receiving PCIA. Two-sample independent t-test was used to compare the results between two groups of patients at the same time points. Repeated measurement analysis of variance (Repeated measures ANOVA) with Bonferroni correction were used for within-group comparisons. *Group K versus Group S (*P*< 0.05). Group K, esketamine group; Group S, sufentanil group. POD, postoperative day; IL-6, Interleukin-6; TNF-α, Tumor necrosis factor-α; BDNF, brain-derived neurotrophic factor.

No significant differences were observed in the serum levels of IL-6, TNF-α, and BNDF between the two groups prior to surgery (all *P*> 0.05). The IL-6 and TNF-α concentrations in group S on POD 1 and POD 2 were numerically higher than those in group K, although with no significant differences (S2 Table). The serum levels of BDNF in group K were significantly higher than those in group S on POD 1 (*P*= 0.046), but no significant differences were observed between the two groups on POD 2 (*P*= 0.593). According to repeated measures ANOVA, no statistically significant differences were observed for IL-6, TNF-α, and BDNF across the repeated measures factor (time) (IL-6: *F* = 0.465, *P* = 0.635; BDNF: *F* = 2.922, *P* = 0.077; TNF-α: *F* = 1.320, *P* = 0.285). The interaction between the repeated measures factor and the grouping factor (times*group) was not statistically significant (IL-6: *F* = 0.574, *P* = 0.573; BDNF: *F* = 3.415, *P* = 0.053; TNF-α: *F* = 0.486, *P* = 0.552). The changes in serum levels of IL-6, TNF-α, and BNDF between the two groups are shown in Fig 4.

## Discussion

In this study, we demonstrated that esketamine-based PCIA resulted in a significant reduction in postoperative pain, promoted early recovery, and provided short-term emotional improvement in patients undergoing VATS lobectomy.

We found that PCIA with 1.5 mg/kg esketamine could reduce the postoperative pain intensity both at rest and during coughing or movement, without the use of opioids. Our results also showed that the SF-MPQ sensory scores, PPI scores, VAS-rest scores, and VAS-movement scores in the esketamine-based PCIA were lower than those in the sufentanil-based PCIA on POD1 and POD 2. Additionally, the total PCIA consumption dosage and the total number of rescue analgesia in the esketamine-based PCIA were significantly less than those in the sufentanil-based PCIA. Additionally, the total PCIA consumption dosage and the total number of rescue analgesia in the esketamine-based PCIA were significantly less than those in the sufentanil-based PCIA. In this study, the analgesic formulas for both groups contained identical ratios of flurbiprofen axetil and dexmedetomidine, thereby minimizing potential bias caused by other drugs. Considering that corticosteroids may affect postoperative pain perception, our study avoided the use of hormonal drugs, and no patients in either group received hormonal drugs during the perioperative period. Patients who received esketamine-based PCIA experienced less pain, supporting the analgesic properties of esketamine. It is important to note that the lung parenchyma is devoid of pain nerve, and the visceral pleura is mainly dominated by the vagus nerve, making the lung tissue insensitive to pain [14]. Therefore, the pain after thoracotomy is primarily attributed to damage to the skin, muscle, and parietal pleura; the contraction of muscles, ligaments, and intercostal nerves; and pleural irritation caused by the placement of a thoracic drainage tube and potential rib fracture [15]. Sufentanil acts on μ-opioid receptor, with little effect on neuroplasticity or reversal of central sensitization, as well as it can relieve the pain at rest, but with little effect on exercise-related pain [16]. Esketamine changes neuroplasticity and mitigates central sensitization [17]. Cheng et al. showed that intraoperative esketamine improved the postoperative pain score at rest and during coughing within 48 h in patients undergoing VATS. Nonsteroidal anti-inflammatory drug (NSAIDs) have anti-inflammatory effects, and in combination with esketamine, they can relieve incision pain and chest wall inflammation [18]. Additionally, the local high concentration of nitric oxide (NO) has been shown to aggravate incision pain [19]. Esketamine has an analgesic effect by inhibiting NO synthase [20,21]. Therefore, compared with sufentanil, esketamine-based PCIA has significant analgesic effects in patients undergoing thoracotomy, which may be related to the ability of esketamine to reduce pain hypersensitivity and chest wall inflammatory stimulation.

The postoperative SF-MPQ affective score of patients receiving esketamine-based PCIA was found to be lower than that of patients with sufentanil-based PCIA, indicating that the perioperative infusion of esketamine can significantly improve the emotional state. Esketamine has been shown to relieve depressive symptoms [22]. Esketamine exerts its antidepressant effects by acting on the anterior cingulate cortex, posterior cingulate cortex, prefrontal cortex, and hippocampus, leading to an increase in the release of glutamate, triggering the activation of the α-amino-3-hydroxy-5-methylisoxazole-4-propionate (AMPA) receptor, stimulating the secretion of BDNF as well as the secretion and activation of mammalian target of rapamycin (mTOR) signaling, thereby producing antidepressant effects [23]. In this study, patients treated with esketamine had a significantly lower postoperative PHQ-9 score than those with sufentanil. Depression has been shown to be closely related to pain, and they were comorbid, sharing neurobiological mechanisms [24]. The analgesic effects of esketamine have also been demonstrated to be beneficial in cases of depression. Jiang et al. [25] administered 0.5 mg/kg ketamine during anesthesia induction for orthopedic surgery, and reported that the postoperative VAS score decreased and the PHQ-9 scores improved. These findings indicate that esketamine can improve patients’ emotional state by relieving pain and depression.

In this study, the EQ-5D score of patients with esketamine-based PCIA was significantly higher, and the time of out-of-bed mobilisation was shorter than those with sufentanil-based PCIA. Early mobilisation is beneficial for pulmonary function recovery in patients undergoing VATS and reduces the incidence of pulmonary complications. Furthermore, a reduction in opioid consumption may reduce opioid-related AEs, such as nausea, vomiting, and respiratory depression. Lower VAS-movement scores and fewer opioids facilitated patients’ early expectoration and mobilisation, which was one of the important reasons why the esketamine-based PCIA group had higher EQ-5D scores. Out-of-bed mobilization earlier, postoperative recovery quality higher. Patients and the observer were more satisfied with esketamine-based PCIA, which may be related to the good analgesia, the emotional benefits and the mild adverse reactions.

Opioids are effective in the management of moderate to severe postoperative pain; however, opioid-related AEs increase patient mortality [26]. The European Society of Thoracic Surgeons has recommended NMDA antagonists to reduce postoperative pain after thoracotomy [27]. In this study, the incidence of nausea and vomiting in patients with esketamine was significantly less than those with sufentanil (10% *vs.* 32.5%). Although one patient had postoperative sedation and one patient experienced nightmare during esketamine-based PCIA, the total incidence of AEs in the esketamine-based PCIA was lower than that in the sufentanil-based PCIA (15% *vs.* 35%). Moreover, no serious psychogenic AEs associated with esketamine occurred. Patients who experienced postoperative sedation had an OAA/S score of 4, and no other comorbid adverse effects were observed. The sedative effect disappeared after the discontinuation of esketamine-based PCIA. One patient receiving esketamine-based PCIA reported a nightmare in which she was on a train that traveled smoothly through the air and didn’t land until she awoke, with no horror factor present. In contrast, another patient receiving sufentanil-based PCIA also experienced a nightmare in which he dreamed some horrible animals and gruesome murder scenes. Given the absence of opioids in PCIA pump, the use of esketamine may further reduce the addiction caused by opioids. To date, there are no reports on the use of esketamine in PCIA for postoperative analgesia in patients undergoing VATS lobectomy. Our preliminary study found that 3.0 mg/kg ketamine- based PCIA relieved postoperative pain in burn patients and improved the quality of analgesia [28]. Given that the analgesic potency of esketamine is approximately twice that of racemic ketamine, 1.5 mg/kg estaketamine was used in this trial. After the end of the trial, we calculated the dosage of esketamine-based PCIA used by patients was 0.33–1.67 μg/kg/min, which was equivalent to 0.15–0.83 μg/kg/min ketamine, lower than the common ketamine dose of 2–5 μg/kg/min [29]. Min et al. reported that adding 2.5 mg/kg esketamine to 100 ml PCIA was an effective postoperative analgesic regimen [30]. Therefore, postoperative analgesia with 1.5 mg/kg esketamine-based PCIA may be safe and effective.

Furthermore, this study preliminarily explored the analgesic and antidepressant partial mechanisms of esketamine. The anti-inflammatory effect of esketamine on analgesia was demonstrated in this study. IL-6 and TNF-α concentrations are well-established biomarkers for inflammation [31,32]. Dale et al. [33] showed that intraoperative ketamine reduced postoperative inflammatory reaction. In this study, compared with the continuous infusion of sufentanil, esketamine further reduced postoperative IL-6 concentration. Zhao et al. found that 0.3 mg/kg ketamine reduced the serum IL-6 and TNF-α levels, thereby reducing postoperative inflammatory response in patients undergoing laparoscopic radical resection [34]. Consistent with these findings, our study showed that postoperative continuous use of esketamine maintained low levels of serum IL-6 and TNF-α, indicating that continuous infusion of esketamine may provide sustained anti-inflammatory effects.

This study showed that esketamine-based PCIA significantly decreased postoperative PHQ-9 scores, and increased serum BDNF levels. Notably, the serum BDNF concentration on POD 1 and POD 2 were higher than the preoperative baseline. BDNF plays a role in the proliferation, differentiation, axon growth, and synapse formation of neurons and glial cells, thereby affecting synaptic transmission and neuroplasticity [35]. Serum BDNF levels are closely related to depressive symptoms [36]. Ketamine blocked NMDA receptors on postsynaptic neurons and inhibited eEF2 kinase, which increased the translation of BDNF. Furthermore, Ketamine indirectly activated AMPA receptors and increased BDNF levels in the brain through ketamine’s metabolites (2R, 6R)-HNK or (2S, 6S)-HNK [24]. It has been established that BDNF is closely associated with depression, with lower BDNF levels correlating with more severe depressive symptoms [37,38], while increased serum BDNF levels were associated with improvements in depression [36,39]. Liu et al. found that patients undergoing total hysterectomy with moderate to severe depression who received intraoperative infusion of esketamine exhibited increased serum BDNF levels alongside reduced scores on the 17-item Hamilton Depression Scale 17 (HAMD-17) [40]. Zheng et al. observed an increase in BDNF levels and a decrease in the Montgomery-Asperger Depression Rating Scale (MADRS) scores in patients with depression after continuous infusion of ketamine [41].

This study has limitations. First, non-competitive inhibition of esketamine on NMDA receptor has been shown to alleviate hyperalgesia and chronic pain [42]. However, due to the early discharge, most patients discontinued the PCIA pump at the end of the study (i.e., upon discharge). Therefore, we did not follow up on the pain after PCIA discontinuation, post-discharge and long-term pain. In the future, we will further explore the clinical impact of esketamine in other patients, other surgeries, and chronic pain. Second, the primary outcome measure of this study was the SF-MPQ scores reflecting patients’ pain conditions between the two groups. Analyses of patients’ perioperative depression and quality of life between the two groups, and changes within each group, were exploratory analyses. The exploratory results are provided for reference only, despite undergoing multiple testing correction. Furthermore, the sample size of patients enrolled was small, necessitating further studies with larger cohorts to validate the generalizability of esketamine for postoperative analgesia and build better predictive models. Third, the effects of esketamine on serum IL-6, TNF-α, and BDNF in our study were a preliminary exploration, with only a small number of patient blood samples collected. The small sample size may have contributed to the lack of significant differences in the results. We will continue to explore the effects of esketamine on perioperative serum IL-6, TNF-α, and BDNF levels. Fourth, this study employed deletion methods to handle missing data, which may present potential bias. Multiple imputation methods can more effectively control the bias. Therefore, we generated 20 imputed datasets using SPSS software (version 25.0), treated the imputed data as true data, and randomly selected one dataset to analyze the primary outcome SF-MPQ scores. The results were compared with those from the per-protocol analysis set (patients who completed the entire study process) to assess the robustness of the main conclusions. This demonstrated no difference between the two approaches in primary outcomes. However, future large-scale studies should employ multiple imputation to reduce selection bias. SF-MPQ results after multiple imputation are presented in S3 Table.

## Conclusions

For patients undergoing VATS lobectomy, 1.5 mg/kg esketamine-based PCIA was associated with a good postoperative analgesia, reducing the SF-MPQ score and the levels of IL-6 and TNF-α, improving perioperative depression and the EQ-5D score, and facilitated early postoperative mobilization, without severe AEs observed.

## Supporting information

S1 Table Recovery quality of patients receiving PCIA. (DOC)

S2 Table Serum IL-6/ BDNF/ TNF-α levels. (DOC)

S3 Table Perioperative SF-MPQ score of patients receiving PCIA (multiple imputation approaches). (DOC)

S1 File Study protocol (original version). (DOCX)

S2 File Study protocol (translated version). (DOCX)

S3 File Data Set. (XLSX)

S1 Checklist CONSORT checklist. (DOC)
