# Supplementary material for: Effect of esketamine-based patient-controlled intravenous analgesia on postoperative pain and quality of recovery after video-assisted thoracoscopic lobectomy: A prospective, double-blind, randomized controlled trial
Source: PLoS One. 2026 Jan 27;21(1):e0340864. doi: 10.1371/journal.pone.0340864 (PMC12843546; doi:10.1371/journal.pone.0340864)
Supplement: S1 Table — (DOC) [file pone.0340864.s001.doc]

**S1 Table. Recovery quality of patients receiving PCIA.**

| variables, median (IQR) | Group S  (n = 40) | Group K  (n = 40) | *z* | *P* value |
| --- | --- | --- | --- | --- |
| PHQ-9 score |  |  |  |  |
| Preoperative | 1.5 (1.00–2.75) | 2.0 (1.00–3.00) | -0.96 | 0.337 |
| POD 1 | 3.0 (1.00–4.00)^a^ | 2.0 (1.00–3.00) | -2.599 | 0.009^*^ |
| POD 2 | 2.0 (1.25–3.00)^b c^ | 1.0 (0.00–1.00)^b c^ | -5.14 | ＜0.001^*^ |
| EQ-5D score |  |  |  |  |
| Preoperative | 1.000 (1.000–1.000) | 1.000 (1.000–1.000) | -1.365 | 0.172 |
| POD 1 | 0.432 (0.292–0.597)^a^ | 0.683 (0.558–0.683)^a^ | -4.362 | <0.001^*^ |
| POD 2 | 0.683 (0.591–0.696)^b c^ | 0.782 (0.683–0.887)^b c^ | -4.593 | <0.001^*^ |
| Time to get out of bed; hour | 21.5 (19–26) | 18 (16–20) | -4.069 | <0.001^*^ |

Data are median (IQR). Mann-Whitney U test was used to compare the results between two groups of patients at the same time points. Wilcoxon signed rank-sum test with Bonferroni correction were used for within-group comparisons. Group K, esketamine group; Group S, sufentanil group; PHQ-9, Patient Health Questionnaire-9; EQ-5D, Euroqol-5 Dimension; IQR, interquartile range; POD, postoperative day. Mann-Whitney U test was used to compare the results between two groups of patients at the same time points. Friedman's test and Wilcoxon signed rank-sum test with Bonferroni correction were used to compare within the two groups.

* The difference between the two groups is significant, *P* < 0.05.

^a^ The within-group difference between the pre-operation and POD 1 was significant, with *P* _(Bonferroni correction)_ < 0.05/3 = 0.016.

^b^ The within-group difference between the pre-operation and POD 2 was significant, with *P* _(Bonferroni correction)_ < 0.05/3 = 0.016.

^c^ The within-group difference between the POD 1and POD 2 was significant, with *P* _(Bonferroni correction)_ < 0.05/3 = 0.016.
