# Supplementary material for: Effect of esketamine-based patient-controlled intravenous analgesia on postoperative pain and quality of recovery after video-assisted thoracoscopic lobectomy: A prospective, double-blind, randomized controlled trial
Source: PLoS One. 2026 Jan 27;21(1):e0340864. doi: 10.1371/journal.pone.0340864 (PMC12843546; doi:10.1371/journal.pone.0340864)
Supplement: S2 Table — (DOC) [file pone.0340864.s002.doc]

**S2 Table**. Serum IL-6 /BDNF/ TNF-α levels

| items | Group S | Group K | *Cohen's D* | *P* value |
| --- | --- | --- | --- | --- |
| IL-6 (ng/L), mean (SD) |  |  |  |  |
| Preoperative | 20.54(29.79) | 17.60(31.46) | 0.10 | 0.871 |
| POD 1 | 20.38(19.19) | 15.20(21.74) | 0.25 | 0.671 |
| POD 2 | 21.07(21.27) | 5.73(7.91) | 0.96 | 0.146 |
| BDNF (ng/L), mean (SD) |  |  |  |  |
| Preoperative | 950.41(379.60) | 898.82(234.93) | 0.16 | 0.783 |
| POD 1 | 929.73(225.93) | 1384.67(434.24) | -1.31 | 0.046* |
| POD 2 | 936.62(319.49) | 1050.36(390.61) | -0.32 | 0.593 |
| TNF-α (ng/L), mean (SD) |  |  |  |  |
| Preoperative | 621.93(358.07) | 587.14(707.69) | 0.06 | 0.917 |
| POD 1 | 613.90(407.53) | 485.45(289.48) | 0.39 | 0.543 |
| POD 2 | 1042.05(894.38) | 643.33(378.01) | 0.58 | 0.338 |

Two-sample independent t-test was used to compare the results between two groups of patients at the same time points. Repeated measurement analysis of variance (Repeated measures ANOVA) with Bonferroni correction were used for within-group comparisons. Group K, esketamine group; Group S, sufentanil group.IL-6, Interleukin-6; BDNF, brain-derived neurotrophic factor; TNF-α, Tumor necrosis factor-α; SD, standard deviation; POD, postoperative day.

* Esketamine group versus Control group (*P*<0.05).
