# Supplementary material for: Effect of esketamine-based patient-controlled intravenous analgesia on postoperative pain and quality of recovery after video-assisted thoracoscopic lobectomy: A prospective, double-blind, randomized controlled trial
Source: PLoS One. 2026 Jan 27;21(1):e0340864. doi: 10.1371/journal.pone.0340864 (PMC12843546; doi:10.1371/journal.pone.0340864)
Supplement: S3 Table — (DOC) [file pone.0340864.s003.doc]

**S3 Table . Perioperative SF-MPQ score of patients receiving PCIA (multiple imputation approaches)**.

| Variables, median (IQR) | Group S  (*n* = 42) | Group K  (*n* = 42) | *z* | *P* value |
| --- | --- | --- | --- | --- |
| Total SF-MPQ score |  |  |  |  |
| Pre-operation | 2.0 (1.00–2.25) | 2.0 (1.00–3.00) | −1.382 | 0.167 |
| POD 1 | 8.0 (6.00–10.25) | 5.0 (4.00–7.00) | −3.966 | <0.001^*^ |
| POD 2 | 6.0 (4.00–8.00) | 2.0 (2.00–4.00) | −5.740 | <0.001^*^ |
| Sensory score |  |  |  |  |
| Pre-operation | 0.0 (0.00–1.00) | 0.0 (0.00–1.00) | −0.707 | 0.480 |
| POD 1 | 3.0 (3.00–4.00) | 3.0 (2.00–3.00) | −3.250 | 0.001^*^ |
| POD 2 | 3.0 (2.00–4.00) | 2.0 (1.00–2.25) | −3.456 | 0.001^*^ |
| Affective score |  |  |  |  |
| Pre-operation | 1.0 (1.00–2.00) | 2.0 (1.00–2.00) | −1.243 | 0.214 |
| POD 1 | 2.0 (1.00–3.00) | 1.0 (1.00–2.00) | −2.910 | 0.004^*^ |
| POD 2 | 2.0 (1.00–2.00) | 0.0 (0.00–1.00) | −5.390 | <0.001^*^ |
| VAS-rest |  |  |  |  |
| Pre-operation | 0.0 (0.00–0.00) | 0.0(0.00–0.00) | 0.000 | 1.000 |
| POD 1 | 1.0 (0.75–2.00) | 1.0(0.00–1.00) | −2.860 | 0.004^*^ |
| POD 2 | 1.0 (0.00–1.00) | 0.0(0.00–0.00) | −4.659 | <0.001^*^ |
| VAS-movement |  |  |  |  |
| Pre-operation | 0.0 (0.00–1.00) | 0.0 (0.00–1.00) | −0.587 | 0.557 |
| POD 1 | 2.5 (2.00–3.00) | 2.0 (1.00–2.00) | −3.126 | 0.002^*^ |
| POD 2 | 2.0 (1.00–2.00) | 1.0 (1.00–1.00) | −5.354 | <0.001^*^ |
| PPI |  |  |  |  |
| Pre-operation | 0.0 (0.00–0.00) | 0.0 (0.00–0.00) | −0.842 | 0.400 |
| POD 1 | 1.0 (1.00–2.00) | 1.0 (0.75–1.00) | −3.113 | 0.002^*^ |
| POD 2 | 1.0 (1.00–2.00) | 0.0 (0.00–0.00) | −5.309 | <0.001^*^ |

Data are median (IQR). Mann-Whitney U test was used to compare the results between two groups of patients at the same time points. Group K, esketamine group; Group S, sufentanil group; IQR, interquartile range; SF-MPQ, Short-Form McGill Pain Questionnaire; POD, postoperative day; VAS, visual analog scale; PPI, present pain intensity.

^*^ The difference between the two groups was significant, with a *P* value of < 0.05.
