# Supplementary material for: Effect of esketamine-based patient-controlled intravenous analgesia on postoperative pain and quality of recovery after video-assisted thoracoscopic lobectomy: A prospective, double-blind, randomized controlled trial
Source: PLoS One. 2026 Jan 27;21(1):e0340864. doi: 10.1371/journal.pone.0340864 (PMC12843546; doi:10.1371/journal.pone.0340864)
Supplement: S1 File — (DOCX) [file pone.0340864.s004.docx]

**艾司氯胺酮在胸科患者术后静脉自控镇痛的临床应用：一项前瞻性、双盲、随机对照试验**

**研究负责人：**阎文军

**研究单位：**甘肃省人民医院

版本号：1.0版

**研究目的：**

本研究旨在研究艾司氯胺酮在胸科患者术后静脉自控镇痛中缓解患者疼痛，减轻胸科患者围术期负面情绪的重要作用，阐明艾司氯胺酮在术后静脉自控镇痛中的作用特点。

**研究方案：**

*研究对象：*该研究自2021年12月至2022年5月在甘肃省人民医院麻醉手术科与胸外一科、胸外二科开展。

*纳入标准：*1）接受胸腔镜肺部手术的胸科患者；2）年龄18-65岁；3）ASA分级Ⅰ～Ⅱ级；4）BMI 18-30；5）意识清楚，有基本的阅读理解能力，视力、听力基本正常；6）既往无精神病史或认知功能障碍，能用语言表达其感受；7）自愿参加本研究，并能理解、签署知情同意书，配合完成干预和评估者

*排除标准：*1）对手术及术后镇痛过程中使用药物及辅料过敏或有禁忌者；2）在入选本研究前3 个月内参与其他临床研究；3）心、肝、肾等重要脏器功能障碍者；4）处于妊娠状态；5）既往有精神病史或认知功能障碍者，近期服用精神类药物史；6）不能理解及沟通交流的患者。

*剔除标准：*1）因严重不良反应或其他原因导致镇痛泵使用中断者；2）伴发其他疾病，不愿继续治疗；3）依从性差，无法配合完成干预或不能提供重要评估信息。

**干预措施：**

*试验组*（K组）：去阿片化药物干预PCIA泵方案，艾司氯胺酮 1.5 mg/kg＋氟比洛酚酯250 mg+胃复安50 mg＋右美托咪定1 μg/kg＋0.9%NS配至150 ml。

*对照组*（S组）：阿片类药物干预PCIA泵方案，舒芬太尼1.5 μg/kg+氟比洛酚酯250 mg+胃复安50 mg＋右美托咪定1 μg/kg+0.9%NS配至150 ml。

首次量2 ml，自控给药量2 ml，持续量2 ml/h。

*补救措施：*抢救性镇痛选择口服氨酚羟考酮片

**研究结局：**

*主要终点：*1）SF-MPQ问卷评分；2）PHQ-9问卷评分；3）VAS镇痛评分

*次要终点：*1）补救镇痛：抢救镇痛用药次数；2）恢复质量：EQ-5D问卷评分、下床时间；3）疼痛因子、炎性因子和情绪因子水平测定：术前12h和术后24h、48h的白介素6、TNF-α、脑源性神经营养因子水平检测；4）不良反应：术后镇静、噩梦、恶心和呕吐以及不良反应的发生率。

**伦理说明：**

（1）对术后行艾司氯胺酮PCIA治疗的患者随访，收集 SF-MPQ 问卷评分、VAS 评分、疼痛和炎症因子水平、术后恶心呕吐（PONV）发生次数和不良反应发生次数。若出现轻度不良反应如噩梦、困倦、头晕等则继续观察；恶心呕吐严重者地塞米松补救性治疗；镇痛不足者给予补救镇痛治疗；

（2）购买白介素6、TNF-α、源性神经营养因子试剂盒，采取术前12h、术后24h、48h空腹血，离心保存并于我院八楼实验室采用生化分析仪进行检测。

**数据采集：**

患者术前术后访视均由经过规范培训的独立麻醉医师统一采集，访视数据由科室相应麻醉医生统一管理。

**统计分析：**

根据前期研究结果，以舒芬太尼为基础进行PCIA治疗的患者24小时平均VAS活动评分为2.6±0.8，而以艾司氯胺酮为基础的患者平均评分为2.0±1.0。设定显著性水平ɑ为0.05（双侧），每组需入组约37例患者以提供不低于80%的把握度（1-β）。

正态分布变量以均数（标准差）表示，采用两样本独立t检验进行分析；非正态分布变量以中位数（四分位间距）表示，采用Mann-Whitney U检验进行分析；分类变量以例数（百分比）表示，采用卡方检验或Fisher确切检验进行比较。

**研究团队：**

阎文军 主任医师 甘肃省人民医院

王玲凯 主管护师 甘肃省人民医院

朱 磊 主治医师 甘肃省人民医院

毕蕊蕊 住培医师 甘肃省人民医院

张继强 住培医师 甘肃省人民医院

粟榆茜 住培医师 甘肃省人民医院

刘瑞娟 住培医师 甘肃省人民医院

李丽娟 医师 甘肃省人民医院

许梦君 医师 甘肃省人民医院

研究构思/设计： 毕蕊蕊、阎文军

文献检索： 张继强

临床研究： 许梦君

实验研究： 李丽娟

数据采集： 粟榆茜

数据分析： 张继强、刘瑞娟

稿件准备和编辑：毕蕊蕊
