# Supplementary material for: Effect of esketamine-based patient-controlled intravenous analgesia on postoperative pain and quality of recovery after video-assisted thoracoscopic lobectomy: A prospective, double-blind, randomized controlled trial
Source: PLoS One. 2026 Jan 27;21(1):e0340864. doi: 10.1371/journal.pone.0340864 (PMC12843546; doi:10.1371/journal.pone.0340864)
Supplement: S2 File — (DOCX) [file pone.0340864.s005.docx]

**Effect of esketamine-based patient-controlled intravenous analgesia on postoperative pain and quality of recovery after video-assisted thoracoscopic lobectomy: A prospective, double-blind, randomized controlled trial**

**Principal Investigator:** Wenjun Yan

**Leading Site:** Gansu Provincial Hospital

Version No.: v1.0

**Purpose of the study:**

The aim of this study is to investigate the important role of esketamine in postoperative patient-controlled intravenous analgesia (PCIA) for thoracic surgery patients in relieving patients' pain and reducing perioperative negative emotions, and to elucidate the characteristics of esketamine's role in postoperative patient-controlled intravenous analgesia.

**Study protocol:**

*Subjects:* the study was carried out from December 2021 to May 2022 in the Department of Anesthesia and Surgery with Department of Thoracic Surgery I and Department of Thoracic Surgery II of Gansu Provincial People's Hospital.

*Inclusion criteria:* (1) patients undergoing VATS lobectomy; (2) age 18–65 years; (3) American Society of Anesthesiologists (ASA) I–II; (4) body mass index (BMI) 18–30 kg/m2; (5) clear awareness, normal reading comprehension, vision, and hearing; (6) no history of mental illness or cognitive impairment; (7) voluntary participation in this study and signed an informed consent form.

*Exclusion criteria:* (1) allergies or contraindications to drugs and adjuvants used in perioperative period; (2) participation in other clinical studies within 3 months before this study; (3) pregnancy or alcohol abuse; (4) history of previous psychiatric or cognitive disorders, with recent history of taking psychotropic drugs; (5) inability to communicate with doctors.

*Elimination criteria:* (1) PCIA interrupted because of serious adverse reactions or other reasons; (2) other illnesses developed during the trial; (3) poor cooperation or failure to provide critical assessment data.

**Intervention:**

*Experimental group* (Group K): esketamine 1.5 mg/kg + flurbiprofen axetil 250 mg + metoclopramide 50mg + dexmedetomidine 1 μg/kg + 0.9% NS dispensed to 150 ml.

*Control group* (group S): sufentanil 1.5 μg/kg + flurbiprofen axetil 250 mg + metoclopramide 50mg + dexmedetomidine 1 μg/kg + 0.9% NS dispensed to 150 ml.

Initial volume 2 ml, self-administered volume 2 ml, continuous volume 2 ml/h.

*Remedy:* salvage analgesic choice of oral one tablet of oxycodone and acetaminophen as rescue analgesia.

**Endpoints:**

*Primary endpoints:* 1) SF-MPQ questionnaire score; 2) PHQ-9 questionnaire score; 3) VAS analgesic score

*Secondary endpoints:* 1) remedial analgesia: number of medications used for rescue analgesia; 2) quality of recovery: EQ-5D questionnaire scores, time out of bed; 3) measurement of levels of pain factors, inflammatory factors, and mood factors: testing of levels of serum interleukin 6, tumor necrosis factor-α, and brain-derived neurotrophic factor at 12h preoperatively and at 24h and 48h postoperatively; 4) adverse reactions: postoperative sedation, nightmares, nausea, and vomiting and incidence of adverse reactions.

**Ethical notes:**

(1) The SF-MPQ questionnaire scores, VAS scores, pain and inflammatory factor levels, the number of occurrences of postoperative nausea and vomiting (PONV), and the number of occurrences of adverse reactions were collected in the follow-up of the patients who underwent postoperative treatment with postoperative patient-controlled intravenous analgesia. In case of mild adverse reactions such as nightmares, drowsiness, dizziness, etc., observation was continued; dexamethasone remedial treatment was given for severe nausea and vomiting; remedial analgesic treatment was given for inadequate analgesia;

(2) Purchase serum interleukin 6, tumor necrosis factor-α, and brain-derived neurotrophic factor kits, take 12h preoperative, 24h postoperative, and 48h fasting blood, centrifugal and preserved and tested in the laboratory on the eighth floor of the hospital using a biochemical analyzer.

**Data collection:**

Patients' preoperative and postoperative visits were uniformly collected by independent anesthesiologists with standardized training, and the visit data were uniformly managed by the corresponding anesthesiologists in the department.

**Statistical analysis:**

According to the results of the preliminary study, the 24-h VAS-movement score was 2.6 (0.8) in patients receiving sufentanil-based PCIA, and was 2.0 (1.0) in those receiving esketamine-based PCIA. Assuming α = 0.05 (two-tailed) and β = 0.2, the required sample size for each group was calculated to be 37 patients.

Normally distributed variables were presented as mean (SD) and analyzed using a two-sample independent t-test. Non-normally distributed variables were reported as median (IQR) and analyzed using the Mann-Whiney U test. Categorical variables were expressed as number (percentages) and compared using the Chi-square or Fisher’s exact test.

**Research Team:**

Study conception/design: Bi Ruirui and Yan Wenjun

Literature search: Zhang jiqiang

Clinical studies: Xu Mengjun

Experimental studies: Li Lijuan

Data acquisition: Su Yuxi

Data analysis: Zhang Jiqiang and Liu Ruijuan

Manuscript preparation and editing: Bi ruirui
